# Supplementary material for: Effect of School-Based Food and Nutrition Education Interventions on the Food Consumption of Adolescents: A Systematic Review and Meta-Analysis
Source: Int J Environ Res Public Health. 2022 Aug 24;19(17):10522. doi: 10.3390/ijerph191710522 (PMC9518323; doi:10.3390/ijerph191710522)
Supplement: Supplementary file 1 [file ijerph-19-10522-s001.zip › ijerph-1795094-supplementary.pdf]

Supplementary Material

	Randomization process	Deviations from intended interventions	Missing outcome data	Measurement of the outcome	Selection of the reported result	Overall Bias
Amani et al. (2006)	?	+	+	+	?	!
Amaro et al. (2006)	?	+	+	+	?	!
Bessems et al. (2012)	+	+	+	+	+	+
Birbaum et al. (2002)	+	+	+	+	?	!
Bjelland et al. (2015)	?	+	+	+	?	!
Bukhari et al. (2011)	?	+	+	+	?	!
Cunha et al. (2013)	+	+	+	+	+	+
Dzewaltowski et al. (2009)	+	+	+	?	+	!
Forneris et al. (2010)	+	+	+	+	+	+
Francis et al (2010)	+	+	+	+	+	+
Ghaffari et al. (2019)	?	+	+	+	?	!
Gratton et al. (2007)	?	+	+	+	?	!
Gray et al. (2015)	?	+	–	+	+	–
Haerens et al. (2006)	–	+	+	+	?	–
Hassapidou et al. (1997)	?	+	–	+	?	–
Hoppu et al. (2010)	?	+	+	+	+	!
Ickovics et al. (2019)	+	+	+	+	+	+
Lytle et al. (2004)	?	+	+	+	?	!
Martens et al. (2008)	?	+	+	+	+	!
Mihas et al. (2009)	+	+	+	+	?	!
Nicklas et al. (1998)	+	+	+	+	+	+
Ochoa-Avilés (2017)	+	+	+	+	+	+
Rees et al. (2010)	+	+	+	+	?	!
Wang et al. (2015)	?	+	+	+	?	!

+ Low risk
? Some concerns
– High risk

Figure S1. Results of each domain and the overall bias of the included articles by Cochrane risk-of-bias tool for randomized trials (RoB 2).

Table S1. Details of school-based intervention studies.

First author, year, country	Participants	Study name and Intervention description.	Main results	Analyses
Amani, 2006. Iran.	60 adolescent girls (30 subjects from each group). Range aged 12–16 years.	The interventional study was undertaken to explore whether a focused nutrition education campaign could improve the nutritional practices, iron status, and hematologic indices of healthy adolescent girls. The education group received nutritional education on dietary sources of iron, iron availability, and the signs and consequences of iron deficiency and anemia via face-to-face group discussion sessions and simple pamphlets; the Control Group did not receive any nutritional education during the 2-month study period. The study groups had no contact with each other. Subjects were asked to follow their usual lifestyle patterns and were informed about the purpose of the study before the start. Fasting blood samples (5 mL) were taken from each subject pre- and post-intervention.	There were no statistically significant differences in any baseline characteristics between the two groups. Scores for nutritional knowledge and practices of the education group were significantly higher after two months compared with the baseline (31.4 ± 6 vs. 24.3 ± 5.9 points, $p < .001$, and 31.2 ± 5 vs. 28.4 ± 5.7 points, $p < 0.05$, respectively). The scores in the Control Group showed no significant changes from baseline to 2 months. The food-frequency scores were elevated in the education group (28.4 ± 5.7 vs 31.2 ± 5, $p < 0.05$) but the control group had a non-significant fall in its scores after the campaign (31.2 ± 8 vs 29 ± 6.5, $p < 0.10$).	Independent and paired t-tests.

Amaro, 2006. Italy.	IG - 153 children (11–14 years old; 78 males, 75 female). CG: (55 male, 33 female).	**Kalèdo (board-game)** The intervention objective was to test the efficacy of Kalèdo (board-game) on changes in nutrition knowledge and dietary behavior. For 24 weeks, group treatment was involved in 15–30 minute-long play sessions once a week. The control group (CG) did not receive any play sessions with Kalèdo.	Children playing Kalèdo showed a significant increase in nutrition knowledge ($p<0.05$) and in weekly vegetable intake ($p<0.01$) concerning the control. Mixed model ANCOVA showed a significant difference between the treated group and CG at post-assessment [$F(1,14)=21.2$; $p<0.01$] for the variable vegetable intake. The adjusted mean number of servings per week was 3.7 (95% CI 3.5 to 4.1) for the treated group and 2.8 (95% CI 2.4 to 3.3) for the CG. A posteriori power analysis showed that, due to the present experimental design and sample size, the detectable difference in the number of weekly vegetable servings with type I error rate of 0.05 and 80% power was 0.7.	Mixed model ANCOVA.
Bessems, 2012. The Netherlands.	13 experimental schools, 1117 students, and 11 control schools, 758 students. Mean age of 12.9 years.	**Krachtvoer program.** This is an updated version of the program. The Krachtvoer program to aim at achieving behavior change based on principles from behavior change theories. The Krachtvoer program aims at increasing the consumption of fruit, achieving a daily healthy breakfast, and decreasing the consumption of fats by replacing high-fat snacks with non-fat or low-fat snacks. Teachers from experimental schools implemented the Krachtvoer lessons over a period of eight weeks between September to December 2008. Control schools carried out the usual nutrition education curriculum aimed at increasing knowledge in the same period and postponed Krachtvoer implementation for one year.	Short- and longer-term favorable intervention effects were found for fruit frequency and yesterday's fruit consumption (servings a day). Experimental condition uncorrected mean (SD), T0 = 0.98 (0.80), T1 =1.13 (0.81); Control condition uncorrected mean (SD), T0 = 1.11(0.92), T1 =1.01 (0.85). β short-term effect = 0.048 (0.023-0.053), ($p<0.001$). β longer-term effect = 0.033 (0.017-0.048), ($p<0.001$). Reported intervention effects were corrected for a random intercept of measurement, student, and class, and the fixed factors of gender, year, educational track, socio-economic positions, and ethnicity. No intervention effects were	Mixed linear and mixed logistic regression analyses.

			found for the breakfast frequency item or the percentage of students who had consumed breakfast that morning. The outcome variables regarding snack frequency and yesterday's snack consumption did not show any effects. Some favorable effects were revealed regarding the categories of snacks consumed, including short- and longer-term effects on sweets consumption and short-term effects on the consumption of savory snacks, ice–creams, and fried snacks. The intervention might be more effective among an older age group.	
Birnbaum, 2002. The USA.	Students in middle and junior high schools. Env +curr + peer, n = 226. Env + curr, n = 677. Env only, n = 845. Control, n = 1,755.	**Teens Eating for Energy and Nutrition at School (TEENS).** The intervention aimed to increase fruit and vegetable intake and decrease the fat intake of low-income young adolescents to reduce their future risk of cancer. The intervention duration was 2 years, but this reports only on the effects of the seventh-grade intervention (1-year period). The intervention consisted of 10 curriculum sessions, informed by social cognitive theory and developed by using a systematic program planning approach. Four incremental exposures were possible: (1) control group, (2) school environment interventions only, (3) classroom curriculum plus school environment interventions, and (4) peer leaders plus classroom curriculum plus school environment interventions: highest exposure. Peer leaders helped teachers deliver the classroom intervention by leading small-group activities and discussions.	Patterns suggesting dose response were observed, with peer leaders reporting the largest increases in fruit, vegetable, and lower fat food consumption. Students exposed to classroom plus environment interventions also improved, whereas students exposed only to school environment interventions showed trends toward choosing lower fat foods and declining fruit intake, and no change in vegetable intake. Changes in self-reported past-year mean daily servings of fruits and vegetables, pre-post seventh-grade intervention, by exposure group - Env only - baseline 4.76(0.04), post-intervention 4.44(0.04), NS; Env + curr - baseline 4.51(0.04), post-intervention 4.95(0.04), p. < 0.1; Env +curr + peer - baseline 4.88(0.06), post-intervention 5.80(0.05), p. < 0.05.	Multiple logistic regression of dropout (yes/no). Multilevel (mixed) regression analyses

			Control students' choices remained stable. Control group - baseline 4.76(0.03), post-intervention 4.80 (0.03), NS.	
Bjelland, 2015. Norway.	Intervention group (IG)- n 498, mean age (SD) 11.2 (0.3); Control Group (CG) – n 898, mean age (SD) 1.2 (0.3).	**HEIA (HEalth In Adolescents) Study.**<br>Intervention aimed to determine if a multi-component health promotion intervention targeting 11–13-year-olds, for 20 months, influenced their consumption of fruit, vegetables and sugar-sweetened beverages.<br>The multicomponent approach in the HEIA study included collaboration with school principals and teachers, school-health services and parent committees, while schoolteachers were the key persons to implement the intervention components. The intervention program consisted of a mixture of individual, group and environmental level strategies and activities.	Effects at 20 months assessment of the adolescents in the HEIA study, mean (DS) for Fruit intake, times/week - CG 9.6 (9.1, 10.0); IG 10.9 (10.4, 11.5), p. < 0.00. For Vegetables, times/week - CG 10.5 (10.0, 11.1); IG 10.9 (10.1, 11.6), p. = 0.46.	Mixed-model Poisson regression; mixed-model analysis of variance.

Bukhari, 2011. The USA.	IG, n = 49 and CG, n = 49).	**The Diet for a Healthy Planet with Teen Battle Chefs**<br>The program aims to build high school students' skills related to cooking and growing food while presenting opportunities for students to gain insight into food production and marketing, as well as how environmental and personal factors can create barriers to opportunities for good nutrition<br>FamilyCook Productions developed a daily, 19-week, ninth-grade curriculum to address nutrition-related attitudinal and behavior changes.<br>The intervention strategies included: skill development, experiential learning activities (eg, photovoice, 8 video, neighborhood food assessments) and personal nutrition challenges (students' reflective diaries of changes made and how the changes affected their mood).<br>In-class activities were designed to increase students' knowledge, build skills, and raise students' self-efficacy for preparing healthful snacks and meals and identifying good food choices (eg, freshly grown fruits and vegetables, whole grains, and minimally processed foods) over processed and prepackaged food. Students were asked to reflect on how to improve healthful food options in their school and community environment via menu changes and use of community gardens and farmers markets.<br>Program Maintenance was addressed by creating an ongoing mechanism for teacher training, as teacher attrition	Analysis of variance and t tests demonstrated improvements in food intake based on the 15 nutrition items selected from the Youth Risk Behavior Survey for this survey. There was an overall increase in score of 4.9 points, or 20.4% (p. < 0.01), in the intervention classes compared with 1.6 points, or 5.7%, in control classes (NS). Improved scores correlated with reporting increases in eating vegetables as snacks (r = 0.64, p. < 0.001), preparing healthful snacks for self (r = 0.48, p. < 0.01), and having sit-down meals with family (r = 0.55, p. < 0.004).	A mixed-model analysis of covariance (ANCOVA).

		presents a problem for program sustainability in most inner-city schools.		

Cunha, 2013. Brazil.	A sample size of 444 children (222 in each study arm) of fifth graders. Mean age (sd) – Intervention Group (IG) 11.2 (1.3); Control Group (CG) 11.2 (1.3).	**PAPPAS (Pais, Alunos e Professores para uma Alimentação Saudável).**<br>The aim intervention was to encouraging students to change their eating habits and food consumption over 9 months (from March to November) in the school year 2010. It focused on positive messages related to the intake of water, fruits, rice, and beans. It also included messages regarding eating fewer cookies, sugar-sweetened beverages, and savory snacks.<br>Trained nutritionists gave monthly 1-h sessions in the classrooms.<br>The activities were designed to discourage students from consuming sugar-sweetened beverages and sugar as well as getting them to replace snacks based on processed food (especially cookies) with fresh fruits or healthy homemade food. To reinforce the message of the activities carried out with children during the class intervention's nutritional sessions, a set of messages was sent to the families in the form of illustrated booklets and recipes. The families also received small gifts such as buttons and magnets. In addition, teachers were encouraged to work with the children on the topics addressed in each intervention session. Thus, a set of exercises and suggestions for class work were provided to them.	Measures of compliance using the frequency of food consumption indicated a statistically significant reduction in the frequency of daily consumption of cookies and sodas and an increased frequency of consumption of fruits in the IG, compared with that of the CG.<br>Variation in daily frequency of cookies, sodas, beans and fruits after the nine months intervention, mean (95% IC): Cookies - CG 0.02 (20.04, 0.09); IG 21.35 (20.20, 20.75); β 0.13, p. < 0.001. Sodas - CG 20.08 (20.18, 0.02), IG 20.20 (20.30, 20.11), β 0.13, p. 0.02. Beans - CG 0.00 (20.35, 0.04); IG 0.01 (20.02, 0.05); β 20.01, p. 0.54. Fruits CG 0.10 (20.05, 0.26); IG 0.17 (0.01, 0.34); β 20.16, p. 0.04.	One-way ANCOVA.

Dzewaltowski, 2009. The USA.	815 youth attending control schools, mean (SD) age - 12.40 (0.43); and 767 children attending intervention schools, mean (SD) age 12.36 (0.40).	**Healthy Youth Places (HYP).** The multilevel intervention model was designed to target the development of the personal and proxy agency of adult leaders and youth to build middle school environments (healthy places) that promote fruit and vegetable (FV) and physical activity (PA). Three tiers (levels) of intervention were used to develop the capacity of adult leaders and youth to build middle school environments (healthy places): project level, school level, place level. At the project level, expert staff delivered continuous group staff training intervention to paid school site coordinators from the eight intervention schools. At the school level, adult site coordinators led the delivery of the change team intervention. This tier mirrored the community hub intervention, with the school site coordinator acting as a facilitator and resource for youth-led school advocacy groups, known as "change teams." The change team was the hub of intervention activities at the school. Students were instructed on the place-based environmental planning and logging system and followed a step-by-step process to implement their environmental change efforts. The school change teams created awareness and visibility within their school regarding the importance of physical activity and good nutrition. A video workgroup at each intervention site, provided with a twice-yearly training, a computer, digital video camera, and video editing software, developed site-specific videos that highlighted ways that students could incorporate FV and PA into specific school settings. Place level, places targeted for implementation included the	The majority of sixth graders were not consuming the recommended number of 5 servings of FV per day. Fruits were eaten more often than vegetables. HYP schools did not change in FV but did significantly change in PA compared to control schools. Proxy efficacy to influence school physical activity environments mediated the program effects. Building the skills and efficacy of adults and youth to lead school environmental change may be an effective method to promote youth PA.	Analysis of variance and t tests.

		classroom, school lunch and the after-school program. The goal of the place level interventions was to reach all children in the intervention grade cohort, build their skills and efficacy for environmental change, and offer them the opportunity for exposure to implemented school environmental changes. In the classroom, a seventh and eighth grade curriculum ("Students Building and Promoting Healthy Places") that targeted building the knowledge and skills for environmental change was implemented to help facilitate student leadership.		
Forneris, 2010. The USA.	Of the students (Sixth graders) at baseline or T1 (n = 2120), 86%, (n = 1830) were present for the first follow-up (T2).	**Goals for Health (GFH)** The intervention was peer-led with high school students teaching health and life skills to sixth-grade students. The purpose of this study was to examine the impact of the GFH school-based program on healthy eating outcomes related to self-efficacy, attitudes, knowledge, and behavior, and to examine the impact of quality of program implementation on the above outcomes. A curriculum, which included a leader manual for each peer leader and a student activity book for each participant, was developed for implementing the program. The peer leaders (N = 144) were high school students chosen by school administrators for their academic performance, leadership qualities, and extracurricular involvement. Leaders received 2 days of training from the GFH staff and a half an hour booster training session each week of the program. Following the 2-day training, the high school students taught the program to middle school students on a weekly for 12 consecutive weeks except for a week break for spring vacation. A project staff member was at each school on the day the program was taught to	Results included significant change patterns across the 4 assessment points in the predicted direction for healthy eating-related self-efficacy and fat and fiber knowledge. No significant change patterns were found at follow-up for FV intake (score), mean (SD), IG: T1 = 1.32 (0.02), T2 = 1.30 (0.02). CG: T1 = 1.33 (0.02). T2 = 1.32 (0.02).	Mixed models to perform a random effects meta-analysis technique.

		supervise the peer leaders and to provide feedback following the session.		
Francis, 2010. Trinidad and Tobago.	579 students - 280 in the Intervention Group (IG) and 299 in the Control Group (CG). Age mean (sd) - IG 10.2 (1.0), CG 10.6 (1.2). The analyzes included 248 in the IG and 224 in the CG.	The objective of the intervention was to improve the knowledge, attitudes and behavior of primary-school children towards better dietary and activity habits. The study was conducted from 6 September 2006 to 30 April 2007.<br>The curriculum consisted of lessons on nutrition and physical activity based on Bloom's mastery learning model. The modules incorporated three domains of educational activities: cognitive, affective, and psychomotor. The content of the curriculum engaged the students in the learning process with activities and experiences geared toward broadening their interests.<br>Students took part in activities where they worked in teams to solve comprehension, vocabulary and mathematics questions based on nutritionally related issues. The physical activity lesson was aimed at increasing energy expenditure both at school and at home.	Average reported daily servings of fried foods were significantly lower in the IG than the CG. Fried food servings/d, mean (SD): Baseline - IG 3.0 (3.1), CG 2.4 (2.5), p. = 0.12. Post-intervention - IG 1.7 (2.1), CG 2.2 (2.5), p. = 0.04<br>Snack foods high in fat, sugar and salt (HFSS foods) 502 kJ/d, mean (SD): Baseline - IG 4.6 (4.4), CG 4.5 (5.2), NS. Post-intervention - IG 3.2 (4.1), CG 4.2 (5.5), NS.<br>In addition, there was a significant decline in reported fried food intake in the IG at the post-intervention assessment compared with baseline. In multivariate regression equations controlling for age, gender, BMI and baseline value, intervention was associated with lower intake levels of fried foods, HFSS and sodas ($P<0.05$).	General linear mixed model regression with SAS PROC MIXED.

Ghaffari, 2019. Iran.	308 students (154 subjects in each group), with age range of 13-15 years.	The intervention aim was to improve the pattern of fruit (F) and vegetable (V) consumption among adolescents by employing Albert Bandura's SCT. The study was implemented in 2017. In student-family-school approach, educational and practical interventions are considered at student, family, and school levels.<br>1. Student-based interventions (e.g., lectures in classrooms with questioning and answering; presenting educational PPT slides and pamphlets on F&V to students; preparation of salads and snacks with F&V in the classrooms by students).<br>2. Family-based interventions (e.g. lecturing with emphasis on the role of parents in receiving F&V; face-to-face communication with parents in discussion group).<br>3. School-based intervention (e.g. educational session for all teachers and managers of schools; installing posters containing health messages related to F&V consumption).	No significant difference in the amount of consumed fruit and vegetable (FV) between the intervention and control groups before the intervention whereas the difference was significant between the groups during 2 and 6 months after the intervention (p. < 0.002, <0.106, respectively).<br>The average amount of FV consumption prior to intervention was 3.7 (1.40) and 2.98 (1.38) in intervention and control groups, respectively. Two months after the intervention were 3.39 (1.56) and 2.83 (1.53) in the intervention and control groups; and 6 months after the intervention, they were found to be 3.27 (1.31) and 3.04 (1.11), respectively.	Repeated measures random effects model.
Gratton, 2007. England.	198 children with ages between 11 and 16 years old (mean 13.1 years, SD 1.32)	The objectives of the intervention were threefold: (i) assess the effectiveness of a motivational based intervention on children's actual dietary behavior and intentions to eat five portions of fruit and vegetables a day, (ii) assess the effectiveness of a volitional-based intervention, using the formation of an implementation intention, to try to increase children's actual dietary behavior and intentions to eat five portions of fruit and vegetables a day and (iii) compare the effectiveness of a motivational vs. a volitional based intervention, in increasing children's actual dietary behavior and intentions to eat five portions of fruit and vegetables a day.<br>**Experimental group A:** Volitional intervention, participants in experimental group A were asked to form an	Both interventions were found to increase fruit and vegetable consumption significantly, although only the volitional intervention demonstrated a significant increase in fruit and vegetable consumption over the control intervention.<br>A MANOVA revealed no significant differences between the three groups at baseline (F(10; 382) = 1.76, p =0.07, n = 0.04).<br>Mean score (SD): Volitional - T1 = 2.24 (0.99), T2 = 2.77 (1.25), p <0.001. Motivational - T1 = 2.05 (0.97), T2 = 2.36 (1.18), p <0.001. Control - T1 = 1.95 (1.13), T2 = 1.91 (1.13), NS.	Multivariate linear and logistic regression analyses.

		implementation intention. This involved the children writing down how, where, and when they would eat five portions of fruit and vegetables for the next 7 days. Participants were asked to keep hold of their plans and put them into action during the week. This activity was given after the Theory of Planned Behavior (TPB) questionnaire and food diary at time 1.<br>**Experimental group B:** The motivational intervention aimed to increase participants' consumption of fruit and vegetables to five portions a day, by using a 'health education activity sheet'. This intervention aimed to change children's beliefs, by using two of the steps suggested by Sutton (2002).<br>**Control group (CG)**<br>The CG formed an implementation intention for how, where, and when to complete their homework for the week. The same method was used for the experimental group except for the emphasis was on writing a plan for completing their schoolwork.		

Gray, 2015.<br>The USA.	Intervention Group (IG) - 5 schools, 20 classes and 562 students and Control Group (CG) - 5 schools, 21 classes and 574 students.	**Choice, Control, and Change.**<br>The intervention was a science and nutrition education curriculum designed to impact the energy balance-related behaviors (EBRBs) of high school students: eating more fruits and vegetables, drinking more water, increasing physical activity and decreasing the intake of sweetened beverages and packaged snacks, eating at fast food restaurants and leisure time. Duration of intervention: 2006-2007. The curriculum consisted of 24 classes and was taught by science teachers on most school days over 8 to 10 weeks, between September and December 2006. Control schools received regular science curricula during the same period and received delayed intervention in the spring of 2007. School teachers received 'Professional Development' that consists of 'Workshops' and 'On-Going Support' to ensure that teachers understand and successfully deliver the curriculum.	The intervention resulted in significant changes in targeted energy balance-related behaviors; in particular, students who participated in the curriculum reported fewer sweetened beverages and processed packaged snacks, smaller sizes at fast food restaurants, decreased leisure screen time and increased physical activity compared with control students.<br>Behavioral outcomes by the 'Teacher Implementation' in the 'Choice, Control and Change' project: Fruits and vegetables intakes at meals or for snacks there was no significant difference. Packaged snacks (mean times/week, (95%IC)) - CG 3.5 (3.3, 3.7), Medium teacher implementation 3.1 (2.7, 3.4), High teacher implementation 3.1 (2.9, 3.3), p value for Omnibus test = 0.016. Fast food (mean size (1–4), (95%IC)) - CG 2.0 (1.9, 2.1), Medium teacher implementation 1.9 (1.8, 2.1), High teacher implementation 1.8 (1.7, 1.9), p value for Omnibus test = 0.001.	Analysis of variance test.

Haerens, 2007. Belgium.	Intervention with parental support (n = 5, 1226 pupils), intervention alone (n=5, 1006 pupils) and a control condition (n=5, 759 pupils). The age range of 11–15 years.	The school-based intervention program was developed to promote healthy food choices and physical activity.<br>The food intervention focused on three behavioral changes that were supported by environmental changes:(1) increasing fruit consumption to at least 2 pieces a day;(2) reducing soft drinks consumption and increasing water consumption; and (3) reducing fat intake. The healthy food intervention was designed for implementation by the school staff itself. Therefore, a working group was composed of the principal, the physical education teacher(s) and other involved teachers. The working group received background information and guidelines on how to address the intervention topics. They received an intervention manual and educational material. Every three months a 1-h meeting with the working group and their search team was held to evaluate the implementation and to plan further actions.<br>Duration of intervention: an academic year - September 2003 to June2004.	The intervention was not effective in increasing self-reported fruit intake and water consumption and no positive intervention effects on soft drinks consumption were found. Pre- and post-intake levels, mean (SD) of fruit intake (pieces week): I + P - pre 5.3 (5.3), post 5.4 (5.3); I - pre 4.6 (5.0), post 4.4 (4.7); C - pre 6.5 (5.0), post 6.0 (4.9); NS.	One-way MANOVA.

Hassapidou, 1997. Greece.	73 students as the Intervention Group (IG0, and 53 more students as the Control Group (CG), aged 13-14 years.	The objective is to stimulate the consumption of fruits and vegetables, as a way of preventing diseases, for example cancer. Duration of intervention: from September 1994 until June 1995.<br>The whole program, which was focused on healthy nutrition, stressed the importance of the Greek diet and encouraged high consumption of fruit and vegetables. The traditional Greek diet includes more fruit and vegetables than the five portions a day recommended in many European countries and the USA. Together with the importance of fruits and vegetables and their role in preventing diseases, emphasis was also given in the program to the reduction of saturated fat intake.	The intervention resulted in significant reduced intake of pork [CG 0.4 (1), IG -0.2 (0.8), $p < 0.05$], salami [CG 0.3 (1.4), IG -0.9 (2), $p < 0.01$], and sausages [0.2 (1.4), IG -0.6 (1.5), $p < 0.05$] in boys in the IG compared with those in the CG. Moreover, boys increased their olive oil consumption and decreased their seed-oil intake. Although the program aimed to increase fruit and vegetable consumption, a decrease was observed in the consumption of apples, pears and kiwi fruit after the intervention. This can be attributed, however, to the seasonal changes (winter to spring).<br>The decrease observed in the IG was smaller than in the CG. Boys in the IG increased their consumption of bananas, grapes and freshly squeezed juices, and they deceased consumption of jam ($p < 0.01$) and cake.<br>Girls in the IG likewise reduced significant their consumption of sausages [CG 0.2 (1.1), IG -0.3 (1), $p < 0.05$], cocoa [CG 1.7 (3.8), IG - 0.02 (3.1), $p <0.05$] and animal butter [CG 2.6 (6.4), IG -1.1 (5.3), $p < 0.01$] . They increased their intake of raw vegetables, but it was not significant. Girls also decreased their consumption of apples and pears, due to the change of season, but they increased their consumption of natural juices. Girls, furthermore, decreased their consumption of honey, cake, and cocoa ($p < 0.05$).	Analysis of covariance (ANCOVA).

Hoppu, 2010. Finland.	Intervention Group (IG), 147 students and Control Group (CG), 140 students. Were on average 13,8 years old.	The intervention aim to decrease the intake of sucrose, and increase the intake of fiber and the consumption of fruit and vegetables among secondary-school pupils.<br>The intervention actions focused on the development of a healthy food environment as well as on nutritional education. In developing the food environment, the main target groups were headmasters, teachers and school catering personnel who carried out the actions at schools after discussing with the study personnel. Nutritional education was implemented by the teachers during regular lessons.<br>For nutritional education, various materials providing information on sugar, fiber, fruit, and vegetables were offered for teachers to use, such as illustrative pictures of typical snacks, posters, informative brochures, games, and tests. Teachers were offered ready-planned lessons, but they were also encouraged to use these materials during their normal lessons according to their needs. Duration of intervention: 2007-2008.	The frequency of consumption of rye bread increased (P=0.03) and that of sweets decreased significantly (P=0.006) in the intervention group (IG) compared to control group (CG). Among boys, there were no significant differences between the intervention and CG. The frequency of daily consumption of vegetables decreased among boys and remained the same among girls in both school groups. Along with the intervention, there was some improvement in the quality of snacks. Girls in the IG reported a decrease in their consumption of sweets significantly compared with girls in the CG(P=0.03). Consumption of sugary soft drinks remained constant among boys in the IG, but at the same time increased significantly among boys in the CG (P=0.02). There were no differences in the changes of consumption of bread and fruit as snacks between IG and CG. Energy-adjusted consumption of fruit, including berries (g/MJ) remained constant in IG (change 1 g/MJ), whereas it decreased in CG (change -6 g/MJ, p = 0.04). There was no significant change in the consumption of vegetables.	Linear mixed models.

Ickovics, 2019. The USA.	Group: Students (mean age (sd)) Intervention Group (IG) physical activity (PA)+ Dual: 152 (10.9 (0.70)) IG PA− Nutrition only:152 (10.7 (0.55)) Control Group (CG) PA+: PA only: 178 (10.9 (0.62)) CG PA - Delayed: 113 (10.9 (0.59))	The primary study objective is to assess effectiveness of implementing school-based nutrition and physical activity policies on student BMI trajectories. All schools received $500/year to support a member of the school community (most often teachers) to establish and lead a School Wellness Team. The focus was on different elements of written policy implementation depending on the schools' randomized study condition. Each school was assigned one research staff member who visited the school one to two times per month. Visits typically included meeting with the School Wellness Team, principal, all teachers for the target grade, school cafeteria manager (nutrition condition), and physical education teachers (physical activity condition). Newsletters were distributed triennially to reinforce targeted health messages (e.g., the Rethink Your Drink campaign). Additionally, nutrition interventions included cafeteria-based nutrition promotion to encourage healthy food choices, taste-testing new foods, and providing alternatives for use of food during celebrations. Physical activity interventions included promotion of active transport (walk/bike) to school, integrating physical activity into classroom lessons, and fitness challenges.	Given significant effects for students in schools randomized to the nutrition intervention, changes in dietary behaviors over time were examined. Specifically, at the end of the study (eighth grade), students in schools randomized to nutrition interventions reported consuming fewer unhealthy foods (mean=1.83 [SD=0.11] vs mean=2.23 [SD=0.12], $\beta = -0.19$, p=0.02), and less frequent consumption of sugar-sweetened beverages (37.95% vs 27.18% drank sugar-sweetened beverages on 2 or fewer days in the past 7 days; OR=1.36, p = 0.025) compared with those in schools with no targeted. Students at schools randomized to receive support for nutrition policy implementation had healthier BMI trajectories over time (F = 3.20, p = 0.02), with a greater magnitude over time and cumulatively significant effects 3 years post-intervention ($\beta = -2.40$, p=0.04).	The significance level was set at $p < 0.05$. It did not mention which tests were used in the study.

Lytle, 2004.<br>The USA.	Intervention Group (IG) 1,452;<br>Control Group (CG)1,431.	**Teens Eating for Energy and Nutrition at School (TEENS)**<br>The intervention goal was to increase students' intakes of fruits, vegetables, and lower fat foods. The TEENS intervention was developed using a theory-based approach to program planning.<br>Three channels were selected for delivery of the intervention including classroom, family, and schoolwide components. The classroom component included 10 behaviorally based nutrition education lessons in each of the seventh and eighth grades. Most schools chose to deliver the TEENS curriculum in Family and Consumer Science; however, in some schools, it was offered in health or science class. The regular classroom teacher delivered the lessons after full day training for each grade level.<br>The TEENS family component consisted of three newsletters and sets of behavioral coupons in both the seventh and eighth grades delivered in conjunction with the TEENS curriculum. In seventh grade, students had assignments related to the family newsletters that were turned in as homework.<br>The schoolwide channel included working with district food service directors and local school food service managers and staff to help foster a school environment where a healthy food choice was the easier and more normative food choice.	Despite positive interim results for students randomized to intervention schools (Birnbaum et al., 2002), the positive effects of the intervention were not seen for the primary outcomes at the end of the 2nd year. No significant differences ($p > 0.05$) fruits and vegetables intake between intervention and control in baseline.<br>Post intervention (score mean, difference, 95%CI):<br>Fruits only - GI 2.34, GC 2.40, Difference -0.060 (–0.309, 0.190). NS difference.<br>Vegetables only - GI 1.73, GC 1.70, Difference 0.031 (–0.250, 0.312). NS difference.<br>Positive effects were seen only for a food choice score (suggesting that the students usually choose lower versus higher fat foods) and not for measures of food intake.	ANOVA.

Martens, 2008. Netherlands.	Intervention Group (IG) - 879 students, and Control Group (CG, 734 students. 32% of the students were aged 12 years or younger, 53% were 13 years old and 15% were 14 years or older	**Krachtvoer program**<br>The overall aim of the program was to increase the consumption of fruit and fruit juice, to decrease the consumption of high-fat snacks and to increase breakfast frequency and quality. Before starting the intervention, mapping was carried out to assist in the realization of each stage in the process of development, implementation and evaluation of theory-based and evidence-based health education programs. The program consisted of eight school classes lasting 50 min each. As recommended by intervention mapping, they systematically used the available theories and evidence to make decisions on program goals and target group, the behavior change methods and strategies, the program design, and implementation strategies. Duration of intervention: September 2002 - December 2002.	Fruit frequency (servings per day) - IG T0 = 0.91 (0.81), T1 = 1.10 (0.86); CG T0 = 0.89 (0.75), T1 = 0.96 (0.81); adjusted for age, gender and baseline (β-value for difference between groups at T1) = 0.043, $p < 0.05$ two-tailed.<br>Fixed regression analyses revealed beneficial effects on the behavioral measures relating to fruit intake (as assessed by a food frequency measure and fruit consumption during the previous day), and most behavioral measures related to high-fat snack intake (food frequency measure, and number of snacks and total fat intake from snacks during the previous day).<br>Mixed regression analysis found comparable regression coefficients for the behavioral outcomes, but the effects related to fruit intake were no longer statistically significant.	Hierarchical linear modeling; generalized linear mixed models Poisson regression.

Mihas, 2009. Greece.	Group: Students, age (sd)<br>Intervention Group (IG): 98 students, 13.1(0.8); Control Group (CG): 93 students, 13.3 (0.9).	**VYRONAS (Vyronas Youth Regarding Obesity, Nutrition and Attitudinal Styles) study.** The intervention aim was to evaluate the short-term (15 days) and long-term (12 months) effects of an intervention in school education on health and nutrition in the diet, nutritional intake and BMI. The health and nutrition components of the program were conducted by the class home economics teacher supervised by a health visitor or a family doctor and incorporated 12 h of classroom material during 12 weeks. The intervention program considered the interactions among environmental, cognitive and behavioral factors, for which Social Learning Theory provides the theoretical framework. Classroom modules were designed to develop behavioral capability, expectations and self-efficacy for healthful eating and healthy foods selection. Learning activities were designed to influence expectancies that placed an important value on achieving these behaviours.	Fifteen days after the intervention, a significant increase in poultry, ready-to-eat breakfast cereals and fruit consumption and a significant decrease in red meat consumption were found in the IG. Poultry, meals/week mean (SD) - IG baseline 1.6 (1.8), post 2.1 (1.7), p 0.041. Ready-to-eat breakfast cereals, meals/week mean (SD) - IG baseline 0.5 (0.5), post 0.7 (0.5), p 0.005. Fruit, portions/week mean (SD) - IG 4.7 (3.5), post 5.9 (4.3), p 0.036. Red meat, meals/week mean (SD) - IG baseline 3.1 (2.5), post 2.4 (2.1), p 0.028.<br>There was no significant difference in the consumption frequency of any food category in the CG.	General linear mixed model analyses.
Nicklas, 1998. The USA.	Identified at baseline (spring 1994) - 2,213 students. At follow-up (spring 1997), 81% of the cohort had participated	**Gimme 5.**<br>It aims to increase the consumption of fruits and vegetables by high school students, through a media campaign, classroom workshops, modification of school meals and parental support. It was used to assign 12 schools (six pairs) to intervention or control conditions. Seven other schools served as pilot test sites. The randomization of the 12 schools occurred after the completion of all baseline measurements. The six school pairs were three female, two male and one coed. One school in each pair was randomly assigned to receive Gimme 5 measurements and interventions, while the	The intervention group reported a 14% increase in consumption of servings of fruit and vegetables after two years of intervention, from 2.63 servings at baseline (1994) to 3.00 servings in 1996. This linear increase was not observed in the control group from 1994 to 1996. IG knowledge scores and awareness indicators were significantly higher than those of the CG (p<0.001).<br>Gimrne 5 provided a first model to show that dietary habits of high school students can be	ANOVA.

	in Gimme 5 measurement for four years, and an additional 15% participated	other schools received only Gimme 5 measurements (controls). The participants of the focus group identified three barriers to increased consumption of fruits and vegetables: 1) lack of availability; 2) lack of variety; and 3) inconsistency in taste. The intervention focused on these barriers. The interventions constituted a school-wide media marketing campaign; classroom activities; modification of school meals ("Fresh Choices"); and parental involvement ("Raisin Teens"). The activities in the classroom and the parents were delivered only to the intervention cohort; however, the entire school benefited from the modification of school meals and the media marketing campaign. Duration of intervention: Changes from baseline (spring 1994) to the end of the intervention period (spring 1997).	influenced by positive media messages relative to that age group, increased exposure to a variety of tasty products, and minimal classroom activity.	

Ochoa-Avilés, 2017. Ecuador.	Group, mean age (sd). Intervention Group (IG): 618 adolescents, 12.9 (0.8); Control Group (CG): 612 adolescents, 12.9 (0.8).	**ACTIVITAL** The program aimed at improving the nutritional value of dietary intake, physical activity (primary outcomes), body mass index, waist circumference and blood pressure (secondary outcomes). A needs assessment, including both qualitative and quantitative data, was performed to ensure appropriateness of the program. The data served to define the intervention objectives and strategies using the Intervention Mapping (IM) and the Comprehensive and Participatory Planning and Evaluation (CPPE) approach. This process resulted in the development of the intervention program named ACTIVITAL with the following intervention objectives: adolescents (i) decrease their sugar intake, (ii) increase their daily fruit and vegetable intake, (iii) decrease their unhealthy snack intake, (iv) increase their healthy breakfast intake, and (v) school food kiosks increase the offer of healthy food. Separate matrices of change objectives for adolescents, parents and school staff were generated. The first stage of ACTIVITAL program in the intervention schools was comprised of three activities: (i) participatory workshops with school staff and adolescents to increase ownership of the intervention and to revise the implementation strategy, (ii) introductory workshops on eating healthily with school staff and adolescents, and (iii) the implementation of intervention package one. Stage two only included the implementation of intervention package two. In the control schools, no additional activities other than the existing national curriculum followed in health science	Participants from the IG consumed lower quantities of unhealthy snacks (−23.32 g; 95% CI: −45.25,-1.37) and less added sugar (−5.66 g; 95% CI: −9.63,-1.65) at the end of the trial. Daily fruit and vegetable intake decreased in both the intervention and control groups compared to baseline, albeit this decrease was 23.88 g (95% CI: 7.36, 40.40) lower in the IG. Waist circumference (−0.84 cm; 95% CI: −1.68, 0.28) was lower in the IG at the end of the program; the effect was mainly observed at stage one. Dose and reach were also higher at stage one.	Linear regression models adjusted and linear mixed models.

		lectures were included. Duration of intervention: 28 months. (30min/day).		

Rees, 2010. United Kingdom.	823 girls, Intervention Group (IG) 406 and Control Group (CG) 417. Range age 12–16 years.	The intervention aim was to evaluate the effectiveness of a computer generated tailored intervention leaflet compared with a generic leaf. The computer generated tailored intervention leaflet aimed at increasing brown bread, whole grain cereal, fruit and vegetable intakes in adolescent girls. In total there were 6 visits. 1 each week and after 3 months there were three more visits, 1 each week as well. Participants received three 24-hour dietary recall sheets over three different days at baseline, and again in follow-up three months later to record food and beverage intake from the previous day. An example of a complete dietary leaf was provided as part of the package so participants could see how to register their food correctly. The recalls were completed during a class with the help of the researchers. Approximately 230 statements were developed to allow tailored responses to an individual's questionnaire data by a team of nutritionists and health psychologists based on qualitative interviews with adolescents. These statements were uploaded to a computer program so that leaflets could be automatically produced according to the questionnaire responses. A standard template for the leaflet was produced using color graphics which was extensively piloted first with small groups of teenage girls. This allowed the leaflet to be adjusted to enhance visual appeal and relevance to the sample following feedback. The leaflets and process were then piloted with 248 girls from one school to ensure the leaflet was well received and the process workable. The intervention tried to capture not only the issue of food intake, but to identify criteria such as behavioral beliefs (attitudes, cognitive and affective beliefs), normative beliefs	The IG consumed approximately 0.35 more servings of brown bread weekly than the CG from baseline. Although this change between groups was statistically significant the magnitude was small. For the other foods there were no significant effects of the tailored intervention.	Three different statistical approaches were used: (i) the repeated measurement procedure in the SPSS statistical software package version 15; (ii) the random effect function me of the package name in the R programmin g language; and (iii) the experimental function gamllsNP in R.

		(subjective and descriptive norms), control beliefs (perceived behavioral control) and intention.		

Wang, 2015 China.	188 students, age range 12-14 years, mean (sd) 12.8 (0.45) years.<br>Health promoting school (HPS school) - 62 students; Health Education (HE school) - 65; and control school – 61.	The intervention aim was to demonstrate the effectiveness of health-promoting school (HPS) framework to promoting healthy eating behaviors and nutrition knowledge among Chinese middle school students, their parents and school staff. The three schools randomly selected were randomly assigned to either (i) a holistic intervention school using the HPS framework (HPS school), (ii) a partial intervention school with a modified Health Education curriculum (HE school) or (iii) a school that does not receive either the HPS or HE intervention (control school).<br>The HPS school implemented a wide range of health promotion activities using the HPS approach. The HE school undertook a modified curriculum intervention only; the control school did not receive either the HPS or the HE intervention, but continued with standard school activities and curriculum. The core information component of the interventions in the HPS school and HE school contained the definition and importance of a balanced diet, the functions of the nutrients, nutrient deficiencies, and their effects, how to supplement necessary nutrients reasonably, good hygienic practices, and food safety. The baseline survey was conducted in July 2012, the intervention activities were implemented in pilot schools. Duration of intervention: September to November 2012.	There was a statistically significant difference in the improvement of eating behaviors of students after interventions across all three schools ($p < 0.001$), with students in the HPS school having the largest improvement in eating behaviors (from 3.16 to 4.13), followed by those in the HE school (from 2.78 to 3.54) and in the control school there was a small increase (from 2.64 to 3.02). There was no statistical difference in the improvement of eating behaviors of parents and school staff after interventions across the three schools.	One-way ANOVA.
